# Determinants and prognostic value of echocardiographic first-phase ejection fraction in aortic stenosis

**DOI:** 10.1136/heartjnl-2020-316684

**Published:** 2020-04-28

**Authors:** Rong Bing, Haotian Gu, Calvin Chin, Lingyun Fang, Audrey White, Russell J Everett, Nicholas B Spath, Eunsoo Park, William SA Jenkins, Anoop SV Shah, Nicholas L Mills, Andrew D Flapan, John B Chambers, David E Newby, Phil Chowienczyk, Marc R Dweck

**Affiliations:** 1 Edinburgh Heart Centre, Royal Infirmary of Edinburgh, Edinburgh, UK; 2 BHF Centre for Cardiovascular Sciences, The University of Edinburgh, Edinburgh, UK; 3 BHF Centre of Research Excellence, King's College London, London, UK; 4 Cardiovascular Medicine, National Heart Center Singapore, Singapore; 5 Guy's and Saint Thomas' Hospitals NHS Trust, London, UK

**Keywords:** cardiac magnetic resonance (CMR) imaging, aortic stenosis, valve disease surgery, echocardiography

## Abstract

**Objective:**

First-phase ejection fraction (EF1) is a novel measure of early left ventricular systolic dysfunction. We investigated determinants of EF1 and its prognostic value in aortic stenosis.

**Methods:**

EF1 was measured retrospectively in participants of an echocardiography/cardiovascular magnetic resonance cohort study which recruited patients with aortic stenosis (peak aortic velocity of ≥2 m/s) between 2012 and 2014. Linear regression models were constructed to examine variables associated with EF1. Cox proportional hazards were used to determine the prognostic power of EF1 for aortic valve replacement (AVR, performed as part of clinical care in accordance with international guidelines) or death.

**Results:**

Total follow-up of the 149 participants (69.8% male, 70 (65–76) years, mean gradient 33 (21–42) mm Hg) was 238 029 person-days. Sixty-seven participants (45%) had a low baseline EF1 (<25%) despite normal ejection fraction (67% (62%–71%)). Patients with low EF1 had more severe aortic stenosis (mean gradient 39 (34–45) mm Hg vs 24 (16–35) mm Hg, p<0.001) and more myocardial fibrosis (indexed extracellular volume (iECV) (24.2 (19.6–28.7) mL/m^2^ vs 20.6 (16.8–24.3) mL/m^2^, p=0.002; late gadolinium enhancement (LGE) prevalence 52% vs 20%, p<0.001). Zva, iECV and infarct LGE were independent predictors of EF1. EF1 improved post-AVR (n=57 with post-AVR EF1 available, baseline 16 (12–24) vs follow-up 27% (22%–31%); p<0.001). Low baseline EF1 was an independent predictor of AVR/death (HR 5.6, 95% CI 3.4 to 9.4), driven by AVR.

**Conclusion:**

EF1 quantifies early, potentially reversible systolic dysfunction in aortic stenosis, is associated with global afterload and myocardial fibrosis, and is an independent predictor of AVR.

## Introduction

Aortic stenosis is the the most common indication for valve intervention in the Western world, with a rising incidence due to an ageing population and increasing numbers of potential candidates for intervention.^1 2^ The current treatment paradigm is based on clinical surveillance for symptom development in patients with severe stenosis. However, there is now growing interest in the development of earlier markers of left ventricular (LV) decompensation that might facilitate more sophisticated risk stratification and thus optimise the timing of aortic valve replacement (AVR).

First-phase ejection fraction (EF1) is a novel measure of myocardial function that is easily measured on routine clinical echocardiography.^3^ It represents LV ejection fraction from the time of aortic valve opening to the time of peak aortic flow (corresponding to the time of maximal ventricular contraction), in contrast to overall ejection fraction. The rationale for EF1 is based on the biophysics of myocyte contraction. As a consequence of early myocardial dysfunction, the first period of ventricular contraction is delayed, even though overall ejection fraction may still be preserved. This altered physiology is quantified by EF1 and is of particular relevance in aortic stenosis, where time to peak aortic valve velocity prolongs with increasing stenosis severity. Preliminary data have demonstrated the feasibility of EF1 measurement in aortic stenosis, proposing a cut-off of <25% that is predictive of future AVR, heart failure or death.^4^

In aortic stenosis, impaired ventricular function can reflect afterload mismatch, where ventricular hypertrophy is insufficient to normalise wall stress and preload reserve is exhausted, and/or myocardial fibrosis, which can be related to the chronic effects of aortic stenosis or concomitant coronary artery disease. Echocardiography is the gold standard imaging modality for assessment of haemodynamics and afterload, while cardiovascular magnetic resonance (CMR) can identify myocardial fibrosis before ejection fraction falls.^5^ In this study, we investigate the associations between haemodynamics, myocardial fibrosis and EF1 in aortic stenosis. Furthermore, we examine the associations between EF1 and clinical outcomes.

## Methods

This cohort was described previously.^6^ Briefly, patients with at least mild aortic stenosis (peak aortic velocity of ≥2 m/s) undergoing follow-up at the Edinburgh Heart Centre were prospectively recruited as part of an observational cohort study between March 2012 and August 2014. Major exclusion criteria included other moderate or severe valvular heart disease, significant comorbidities with limited life expectancy, contraindications to gadolinium-enhanced CMR, and acquired or inherited non-ischaemic cardiomyopathies as assessed by history or CMR. Patients underwent clinical evaluation, venous blood sampling for plasma high-sensitivity cardiac troponin I (hs-cTnI, ARCHITECT STAT assay; Abbott Laboratories, Abbott Park, Illinois, USA) and brain natriuretic peptide (BNP; Triage assay; Biosite, San Diego, California, USA) concentrations, electrocardiography, transthoracic echocardiography and CMR. Patients were referred for AVR by the treating cardiologist in accordance with routine practice and contemporary guidelines, with heart team discussion where appropriate.^7^

### Echocardiography

Baseline transthoracic echocardiography was systematically performed in all patients by a dedicated British Society of Echocardiography-accredited research ultrasonographer or cardiologist. Standard measurements were performed and aortic stenosis severity was graded according to contemporaneous American Society of Echocardiography guidelines.^8^ EF1 was retrospectively analysed by two investigators (HG and LF) blinded to patient characteristics and outcomes using EchoPAC (GE Healthcare, Chicago, Illinois, USA). All EF1 measurements were undertaken after the last census date for outcomes. LV volumes were measured using Simpson’s biplane method. EF1 was calculated: (LV end-diastolic volume–LV volume at time of peak aortic flow)/LV end-diastolic volume×100. Valvuloarterial impedance (Zva), a validated and prognostic measure of global LV afterload in aortic stenosis, was also calculated: (systolic blood pressure+mean aortic valve gradient)/stroke volume indexed to body surface area).^9^

### Cardiovascular magnetic resonance

Detailed CMR protocols in this cohort have been described.^6^ All scans were performed on a 3 T scanner (MAGNETOM Verio, Siemens AG, Erlangen, Germany). Image analysis was performed using OsiriX V.4.1.1 (Geneva, Switzerland). Short-axis cine images were used to calculate LV volumes, mass and ejection fraction. Late gadolinium enhancement (LGE) imaging was performed 15 min after administration of 0.1 mmol/kg gadobutrol, was independently assessed as present or absent, and, if present, infarct or non-infarct pattern by two investigators (CC and MD). T1 mapping was performed using the modified Look-Locker inversion recovery sequence.^10^ Native T1, extracellular volume fraction (ECV%) and indexed extracellular volume (iECV=ECV%×LV diastolic myocardial volume indexed to body surface area) were measured. This latter measurement provides an absolute estimate of the extracellular compartment, in contrast to ECV%, which estimates this volume relative to cellular volume.^6 11^ Both ECV% and iECV included non-infarct and excluded infarct LGEs.^11^ Global longitudinal strain (GLS) was measured using CVI V.4.2 (Circle Cardiovascular Imaging, Canada). A fully automated strain analysis was carried out and two-dimensional (2D) GLS was used as the primary strain assessment. Echocardiographic GLS software was not available for this cohort, but measurements acquired on echocardiography and CMR have been shown to correlate well.^12^

### Outcomes

Clinical outcomes, including AVR, were captured from medical records, while deaths were identified through the General Register of Scotland. Post-AVR EF1 was measured as close to 1 year follow-up as possible on research or clinical echocardiograms.

### Statistical analysis

Continuous variables were tested for normality with the Shapiro-Wilk test and are presented as median (IQR) or mean±SD. Non-normally distributed continuous variables were log_2_ transformed. GLS was log_2_ transformed after addition of a constant (greatest GLS+1). EF1 was dichotomised as high (≥25%) and low (<25%).^4^ Univariable linear regression modelling was performed to identify the associations between EF1 and relevant clinical and CMR variables. Covariables identified for inclusion in multivariable models were age (per decade), male sex, Zva, infarct LGE, iECV, hs-cTnI and BNP. These models were constructed with haemodynamics and CMR parameters, followed by the addition of biochemistry and clinical parameters. As this model assessed explored potential mechanistic associations, Zva was included as an integrated measure of function and haemodynamics. Infarct LGE and iECV were chosen due to collinearity between other fibrosis measures. Cumulative event rates were examined using Kaplan-Meier curves for the combined primary endpoint of AVR or all-cause mortality. Assumptions for proportionality were checked and Cox proportional hazards models were constructed to assess the prognostic utility of EF1, with the covariables of age (per decade), male sex, New York Heart Association (NYHA) dyspnoea class, mean gradient, EF1, infarct LGE and iECV. Mean gradient, rather than Zva, was chosen as a more commonly used parameter for this clinical outcome model. Two-sided p values of <0.05 were considered statistically significant. Analysis was performed using R version3.5.0 (R Foundation for Statistical Computing, Vienna, Austria).

### Patient and public involvement

This article was written without patient involvement. Patients were not invited to comment on the study design and were not consulted to develop patient-relevant outcomes or to interpret the results. Patients were not invited to contribute to the writing or editing of this document for readability or accuracy.

## Results

### Patient characteristics

**Figure 1:**
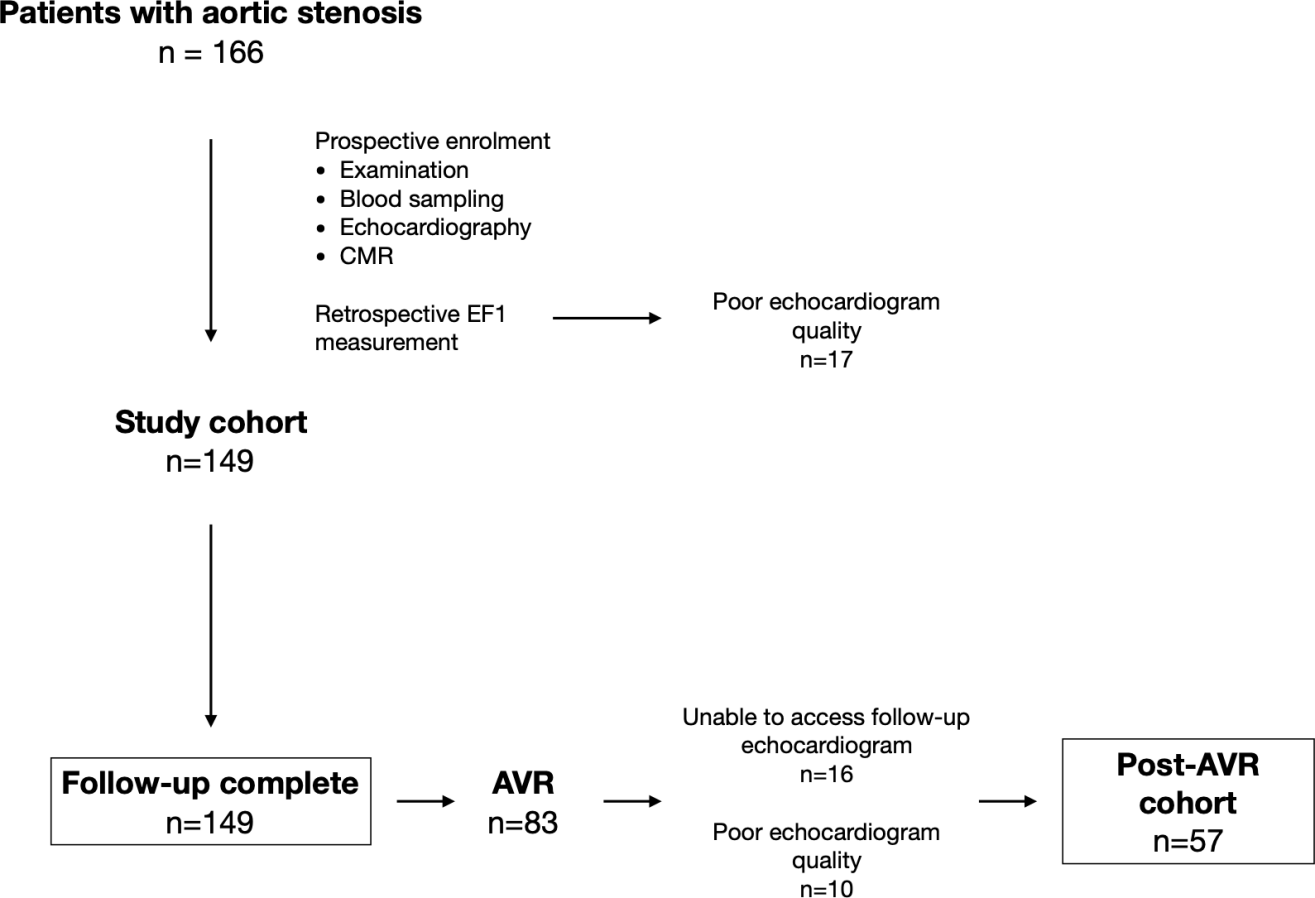
Study flowchart study design demonstrating patient population and investigations at baseline and follow-up. AVR, aortic valve replacement; CMR, cardiovascular magnetic resonance; EF1, first-phase ejection fraction.

**Figure 2:**
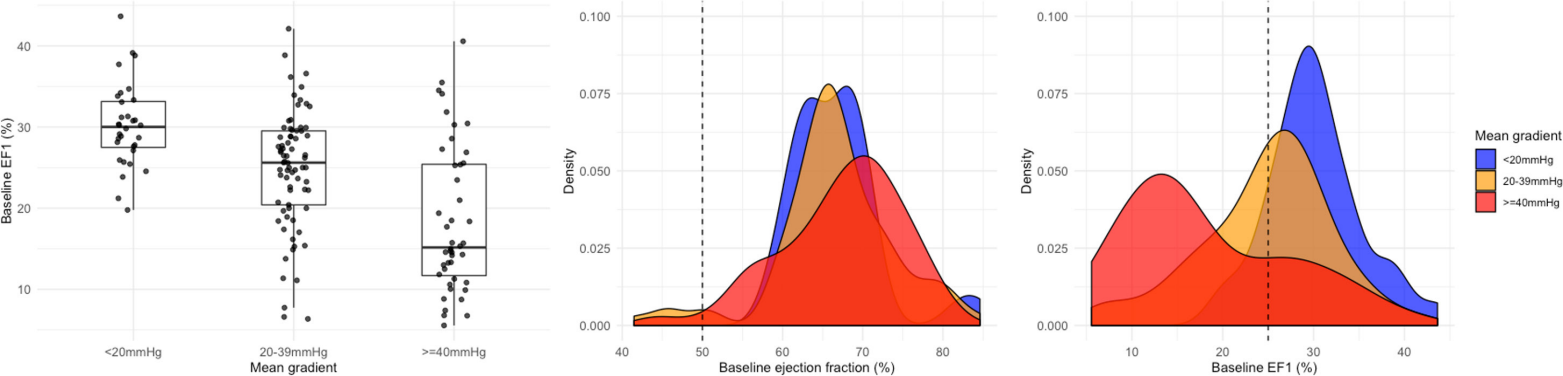
Distribution of EF1 and ejection fraction by aortic stenosis severity box plot demonstrates EF1 according to mean gradient (<20mm Hg: 30 (27–33)%, 20–39 mm Hg: 26 (20–30)%, ≥40 mm Hg: 15 (12–25)%). Density plots demonstrate the distribution of EF1 and ejection fraction among patients with aortic stenosis stratified by mean gradient. The dashed reference lines denote 25% and 50% for EF1 and ejection fraction, respectively. EF1, first-phase ejection fraction.

EF1 was measurable in 149 of 166 (90%) participants. Seventeen were excluded due to suboptimal echocardiographic LV endocardial definition (figure 1); these patients had higher aortic valve gradients than the remaining study cohort (mean gradient 42 (30–54) mm Hg vs 33 (21–42) mm Hg, p=0.006). The study cohort comprised patients with mild (n=34), moderate (n=40) and severe (n=75) aortic stenosis, of whom 67 (45%) had a low baseline EF1 (<25%) (table 1 and online supplementary table 1). Twenty-eight patients were categorised as severe based on aortic valve area of <1.0 cm^2^ with a discordant mean gradient of <40 mm Hg, of whom 4 had an indexed stroke volume of <35 mL/m^2^. Patients with low EF1 were of similar age and had more severe aortic stenosis than those with an EF1 of ≥25% (figure 2). Markers of LV decompensation were more common in those with a low EF1, including worse NYHA class, higher plasma hs-cTnI concentrations and more non-infarct myocardial fibrosis. The prevalence of infarct LGE and coronary artery disease was also higher in patients with a low EF1. Importantly, there was no difference in ejection fraction between the low and normal EF1 groups (67% (62%–71%) vs 67% (63%–70%), p=0.47). Only four patients in the low EF1 group had an ejection fraction of <50% (range 42%–46%), while 13 patients in the low EF1 group had an ejection fraction of <60%.

10.1136/heartjnl-2020-316684.supp1 Supplementary data

**Table 1:** Baseline characteristics

	Overall	EF1≥25%	EF1<25%	P value
n	149	82	67
Age (years)	70.0 (65.0 to 76.0)	70.5 (63.0 to 77.0)	69.0 (66.0 to 75.0)	0.97
Male sex	104 (69.8)	51 (62.2)	53 (79.1)	0.04
Hypertension	102 (68.5)	58 (70.7)	44 (65.7)	0.63
Hyperlipidaemia	67 (45.0)	35 (42.7)	32 (47.8)	0.65
Diabetes	21 (14.1)	14 (17.1)	7 (10.4)	0.36
Coronary artery disease	56 (37.6)	20 (24.4)	36 (53.7)	<0.001
Systolic blood pressure (mm Hg)	148.5 (137.0 to 165.5)	153.0 (140.5 to 169.6)	144.0 (134.5 to 159.0)	0.10
Diastolic blood pressure (mm Hg)	84.0 (77.0 to 92.0)	82.0 (76.5 to 90.0)	84.5 (79.2 to 92.8)	0.21
NYHA				0.005
I	71 (47.7)	48 (58.5)	23 (34.3)
II	49 (32.9)	25 (30.5)	24 (35.8)
III	26 (17.4)	9 (11.0)	17 (25.4)
IV	3 (2.0)	0 (0.0)	3 (4.5)
AV Vmax (m/s)	3.8 (3.2 to 4.3)	3.4 (2.8 to 3.9)	4.1 (3.8 to 4.5)	<0.001
AV mean gradient (mm Hg)	32.9 (20.7 to 41.7)	24.0 (16.3 to 35.2)	38.7 (33.8 to 45.2)	<0.001
AV area (cm^2^)	0.9 (0.7 to 1.1)	1.0 (0.8 to 1.2)	0.8 (0.7 to 0.9)	<0.001
Valvuloarterial compliance (mm Hg/mL/m^2^)	4.0 (3.3 to 4.4)	3.9 (3.2 to 4.3)	4.1 (3.4 to 4.6)	0.18
Indexed LV mass (g/m^2^)	87.0 (73.0 to 99.0)	81.5 (69.2 to 93.0)	95.0 (81.5 to 101.5)	0.001
Indexed stroke volume (mL/m^2^)	47.0 (41.0 to 54.0)	47.0 (41.0 to 53.0)	47.0 (41.0 to 54.4)	0.83
Ejection fraction (%)	66.7 (63.0 to 70.7)	66.7 (63.2 to 70.4)	66.7 (62.4 to 70.9)	0.47
EF1 (%)	25.6 (17.7 to 29.9)	29.6 (27.3 to 32.8)	15.7 (12.2 to 20.9)	<0.001
Global longitudinal strain (%)	−17.9 (−20.1 to −15.4)	−18.0 (−20.7 to −16.0)	−17.7 (−19.3 to −14.7)	0.047
Native T1	1179.0 (1157.0 to 1207.0)	1174.0 (1149.5 to 1201.5)	1188.5 (1165.0 to 1209.2)	0.08
ECV fraction (%)	27.6 (25.6 to 29.1)	27.3 (25.6 to 28.4)	27.9 (25.8 to 30.0)	0.16
iECV (mL/m^2^)	22.3 (18.7 to 26.2)	20.6 (16.8 to 24.3)	24.2 (19.6 to 28.7)	0.002
Infarct LGE	21 (14.1)	5 (6.1)	16 (23.9)	0.004
Non-infarct LGE	36 (24.2)	13 (15.9)	23 (34.3)	0.015
hs-cTnI (ng/L)	6.6 (3.6 to 12.4)	4.9 (3.2 to 9.1)	9.3 (4.3 to 15.3)	0.009
BNP (ng/L)	26.8 (12.4 to 54.2)	23.9 (10.3 to 49.9)	30.0 (14.5 to 71.4)	0.07

AV, aortic valve; BNP, brain natriuretic peptide; ECV, extracellular volume; EF1, first-phase ejection fraction; hs-cTnI, high-sensitivity cardiac troponin I; iECV, indexed extracellular volume; LGE, late gadolinium enhancement; LV, left ventricular; NYHA, New York Heart Association; Vmax, peak aortic jet velocity.

### Clinical characteristics associated with EF1

On univariable linear regression analyses, EF1 was associated with aortic valve mean gradient, Zva, indexed LV mass, native T1, iECV, LGE, GLS and hs-cTnI (see online supplementary table 2). Stepwise multivariable linear regression models using the prespecified covariables (table 2) demonstrated Zva, iECV (both p<0.001) and infarct LGE (p=0.047) to be independently associated with EF1, although there remained substantial unexplained variance (*r* ^2^=0.25, p<0.001). Of note, there was no correlation between EF1 and ejection fraction (Pearson’s *r*=0.14, p=0.10).

**Table 2:** Stepwise multivariable linear regression models for EF1

	Model 1 (*r* ^2^=0.19), n=145			Model 2 (*r* ^*2*^=0.25), n=126			Model 3 (*r* ^2^=0.25), n=126		
	Coefficient	95% CI	P value	Coefficient	95% CI	P value	Coefficient	95% CI	P value
Zva	−0.48	0.75 to 0.20	<0.001	−0.64	0.95 to 0.33	<0.001	−0.66	0.99 to 0.34	<0.001
iECV	−0.49	0.75 to 0.23	<0.001	−0.72	1.06 to 0.37	<0.001	−0.70	1.05 to 0.34	<0.001
Infarct LGE	−0.35	0.63 to 0.07	0.01	−0.33	0.63 to 0.02	0.036	−0.31	−0.63 to 0.00	0.047
hs-cTnI	0.025	−0.06 to 0.11	0.57	0.02	−0.06 to 0.11	0.61
BNP	0.04	−0.04 to 0.11	0.32	0.03	−0.06 to 0.11	0.55
Age per 10 years							0.03	−0.08 to 0.13	0.63
Male							−0.03	−0.26 to 0.21	0.81

BNP, brain natriuretic peptide; EF1, first-phase ejection fraction; hs-cTnI, high-sensitivity cardiac troponin I; iECV, indexed extracellular volume; LGE, late gadolinium enhancement; Zva, valvuloarterial impedance.

### Change in EF1 after AVR

In patients who underwent AVR, we examined the change in EF1 after relief of afterload. Of the 149 patients with baseline EF1 data, 83 (56%) underwent AVR (baseline echocardiogram to AVR 14.9 (2.9–35.5) months). The primary indications for AVR were dyspnoea (n=48), chest pain (n=12), presyncope/syncope (n=9), rapid progression or very severe stenosis (n=7), heart failure (n=3) or asymptomatic LV dysfunction (n=2). One patient was referred due to a positive exercise treadmill test, and one patient underwent AVR to facilitate hip replacement. Sixteen patients did not have post-AVR echocardiograms available, while 10 had echocardiograms of insufficient quality to measure EF1, leaving 57 patients for analysis (AVR to follow-up echocardiogram at 11.2 (6.0–13.1) months).

Overall, EF1 increased after AVR (baseline, 16% (12%–24%), vs follow-up, 27% (22%–31%); p<0.001). A cut-off of ≥3% was then applied as a threshold for improvement based on prior intraobserver variability.^4^ Thirty-nine (68%) patients improved, while 18 (32%) did not. Of these 18 patients, 11 had a low EF1 at baseline (see online supplementary figure 1). Baseline clinical and imaging characteristics in these 11 patients with a fixed low EF1 were similar to the remaining cohort (see online supplementary table 3), including aortic stenosis severity, Zva, EF1 and ejection fraction. However, patients with a fixed low EF1 had a much higher prevalence of infarct LGE (64% vs 9%, p<0.001). On univariable logistic regression analysis, infarct LGE was the only parameter that was associated with a fixed low EF1 (coefficient 2.9, 95% CI 1.3 to 4.5, p<0.001) (see online supplementary table 4).

10.1136/heartjnl-2020-316684.supp2 Supplementary data

### EF1 and outcomes

**Figure 3:**
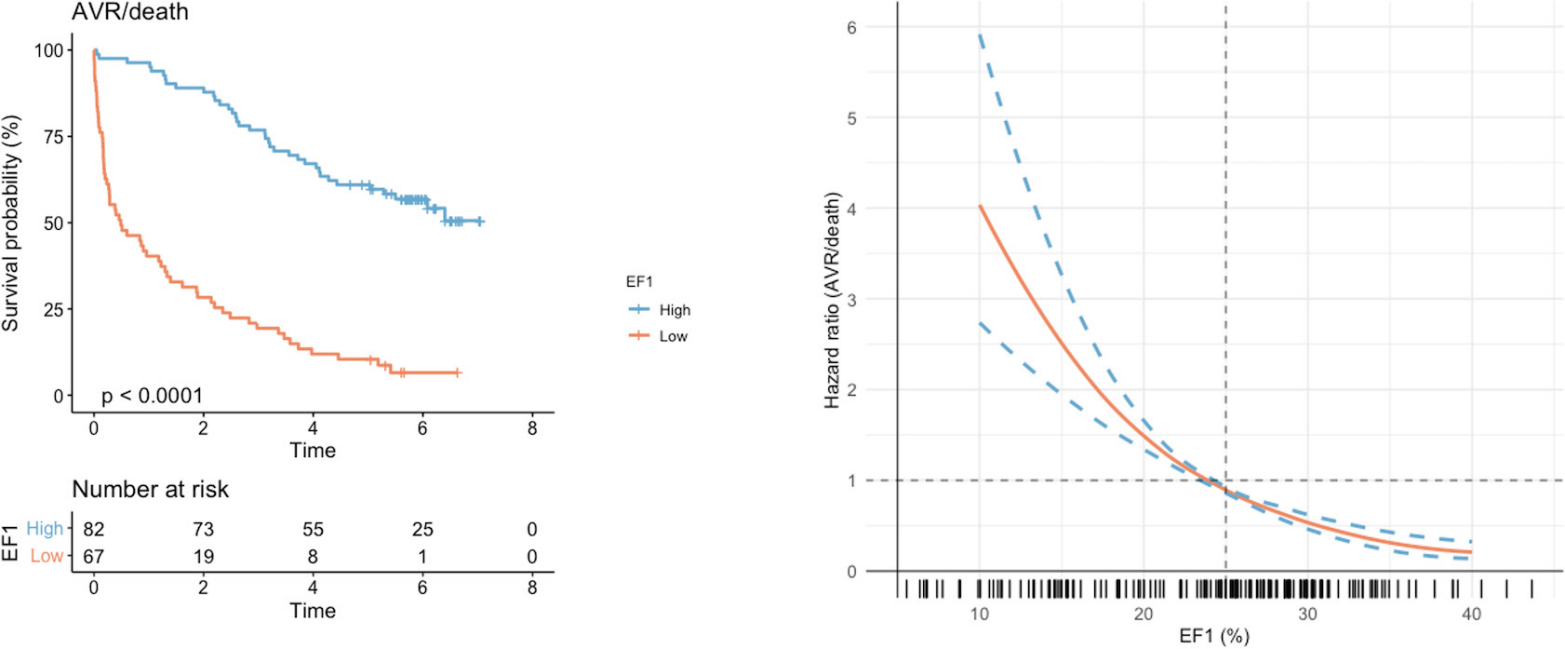
Association between EF1 and outcomes The Kaplan-Meier curve (left) demonstrates unadjusted survival free of AVR or death in the study cohort (n=149), stratified by high (≥25%) or low (<25%) EF1 (p value for log-rank test). The Cox regression curve (bottom right) demonstrates the predicted HR for AVR or death according to EF1, adjusted for age, mean gradient and indexed extracellular volume. The distribution of EF1 in the study cohort is presented in the rug plot below the regression curve. AVR, aortic valve replacement; EF1, first-phase ejection fraction.

**Figure 4:**
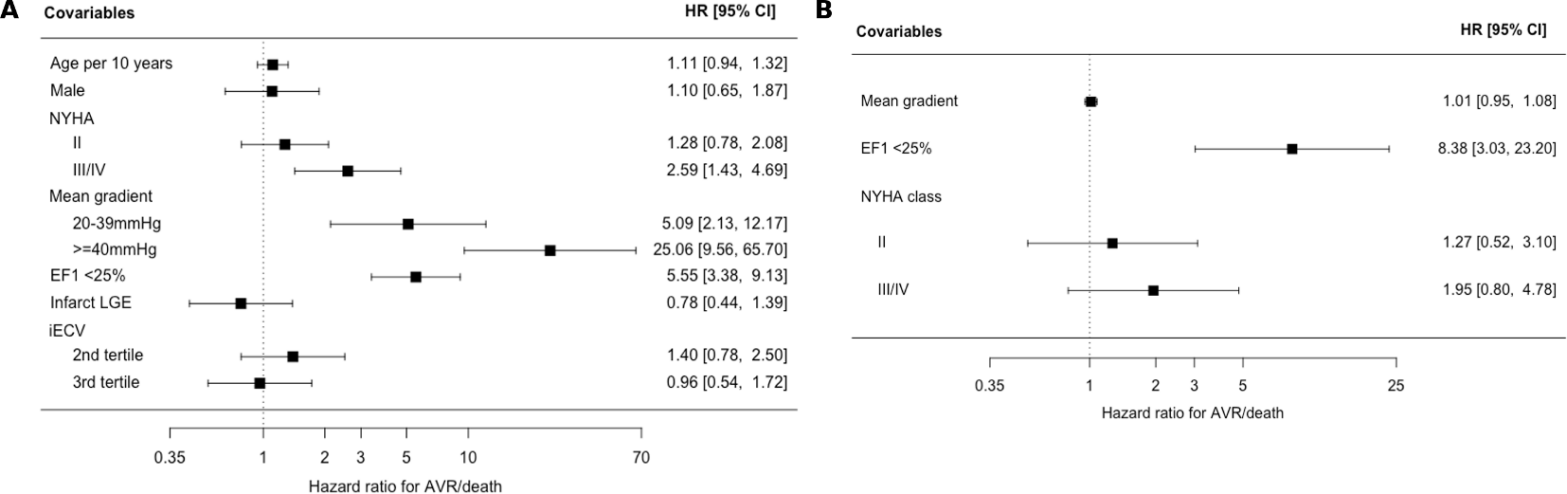
Multivariable predictors of AVR or death forest plots of multivariable Cox proportional hazards models for the composite endpoint of AVR or death. HR scales are presented as logarithmic scales. NYHA was stratified according to class (reference: class I). (A) Model for the whole cohort. Aortic stenosis severity was stratified by mean gradient (<20 (reference), 20–39 and ≥40 mm Hg). iECV was stratified by tertiles (first tertile as reference). (B) Model for patients with a mean gradient of ≥40 mm Hg (n=44; 36 had AVR, 5 died before AVR). The model was restricted to these three variables to avoid overfitting. The mean gradient was analysed as a continuous variable in keeping with the lack of a clear threshold in clinical practice for these patients. AVR, aortic valve replacement; EF1, first-phase ejection fraction; iECV, indexed extracellular volume; LGE, late gadolinium enhancement; NYHA, New York Heart Association.

Patients were followed up for a total of 238 029 person-days. More than double the proportion of patients with a low EF1 reached the primary endpoint of AVR or death, compared with patients with a normal EF1 (93% vs 45%, p<0.001) (table 3 and figure 3). This was driven by AVR (81% vs 35%, p<0.001). Death was numerically higher in the low EF1 group (21% vs 12%, p=0.23), and all five patients who died after AVR had a fixed low EF. The optimal threshold in our cohort for prediction of the primary endpoint was 24% (Youden’s index of 0.55), almost identical to the cut-off of 25% described previously^4^ and applied here (see online supplementary figure 2). There were no differences in other cardiovascular outcomes, although there were few events. Multivariable Cox proportional hazards modelling demonstrated low EF1 to be an independent predictor of the primary endpoint (HR 5.6, 95% CI 3.4 to 9.1) alongside mean gradient and NYHA class (figures 3 and 4A). CMR assessments of myocardial fibrosis were not independent predictors of the primary outcome. Excluding patients with mild aortic stenosis did not alter these associations (see online supplementary figure 3). A limited Cox proportional hazards model in patients with a mean gradient ≥40 mm Hg (n=44; 36 had AVR, 5 died before AVR) again demonstrated EF1 to be an independent predictor of the primary endpoint. Of note, mean gradient and NYHA class were not predictors in this model after adjusting for EF1 (figure 4B). In this subgroup, the optimal threshold for the composite endpoint was 25% (Youden’s index of 0.76).

10.1136/heartjnl-2020-316684.supp3 Supplementary data

10.1136/heartjnl-2020-316684.supp4 Supplementary data

**Table 3:** Outcomes stratified by EF1

	EF1≥25%	EF1 (<25%)	P value
n	82	67	
AVR or death	37 (45.1)	62 (92.5)	<0.001
AVR	29 (35.4)	54 (80.6)	<0.001
Death	10 (12.2)	14 (20.9)	0.23
Cardiovascular death	6 (7.3)	5 (7.5)	1.00
Myocardial infarction	3 (3.7)	4 (6.0)	0.78
Cerebrovascular event	6 (7.3)	4 (6.0)	1.00
Heart failure	5 (6.1)	4 (6.0)	1.00

AVR, aortic valve replacement; EF1, first-phase ejection fraction.

For comparison, we performed an exploratory analysis of GLS and ejection fraction as alternative markers of impaired ventricular function in the whole cohort. On univariable analysis, a reduced GLS was associated with an increased incidence of AVR or death (HR 2.3, 95% CI 1.5 to 3.5). However, after constructing the same multivariable Cox proportional hazards model as used for EF1, GLS was no longer a predictor of the primary endpoint (HR 1.54, 95% CI 0.92 to 2.6). Similarly, ejection fraction was not an independent predictor of the primary endpoint when replacing EF1 in the final model (HR 0.99, 95% CI 0.96 to 1.00).

## Discussion

We have demonstrated associations between EF1 and myocardial disease in patients with aortic stenosis. In particular, we report that EF1 decreases with increasing aortic stenosis severity despite preserved overall ejection fraction, and that this reduction in EF1 is associated with increased global afterload and myocardial fibrosis burden. Of note, a low EF1 is generally reversible after relief of afterload. Finally, we show that EF1 is a powerful predictor of future AVR, independent of mean gradient.

### EF1: rationale and associations

The development of symptoms and adverse events in aortic stenosis are related not only to progressive valve narrowing but also the remodelling response of the left ventricle.^13^ Current guidelines recommend AVR in patients with severe aortic stenosis and symptoms or evidence of LV decompensation - for example, a reduction in ejection fraction or an elevated BNP.^7 14^ However, symptoms are often difficult to assess, while deterioration in ejection fraction and elevation of BNP may occur relatively late. Consequently, there is a clinical need for a sensitive, dynamic biomarker that identifies the onset of early, reversible LV dysfunction. Although CMR assessments of myocardial fibrosis show promise and have prognostic value,^5 15–19^ they require an additional, less accessible scan. In contrast, EF1 can be easily performed on routinely acquired echocardiograms in <10 min without the need for dedicated software or imaging techniques.

EF1 differs from overall ejection fraction. We observed no correlation between these two parameters, with marked reductions in EF1 seen in the context of a preserved ejection fraction. Three measures were independently associated with EF1: Zva, a measure of global LV afterload; iECV, reflecting non-infarct fibrosis; and infarct LGE. This is not surprising, given that these processes are recognised determinants of LV systolic function in aortic stenosis.^20^ Importantly, reductions in EF1 generally improved following AVR, consistent with the reversible nature of afterload mismatch and diffuse fibrosis.^6 11^ The small number of patients with a fixed low EF1 (n=11) had a much higher prevalence of infarct-related, non-reversible replacement fibrosis despite a relatively preserved ejection fraction (66% (53%–69%)).

### EF1: prognosis

We have shown that EF1 is a powerful and independent predictor of AVR or death when using the previously reported threshold of 25%, a cut-off very similar to the optimal threshold in our cohort. This was driven by AVR and was independent of aortic stenosis severity and myocardial fibrosis. Notably, in our cohort, the same independent associations were not seen with CMR 2D-GLS or ejection fraction. GLS in particular is a recognised marker of early ventricular dysfunction, but there was only a small difference in baseline GLS between the high and low EF1 groups. This could reflect the sensitivity of EF1, with changes occurring earlier in the disease course. We were unable to show an independent association between GLS and the primary endpoint, despite this association being demonstrated in other larger cohorts. As such, our findings should be interpreted with caution and in the context of the existing body of literature. Further larger studies of EF1 and GLS to explore their comparative associations and prognostic value would be most interesting. Particularly relevant for future clinical application is the predictive power of EF1 in patients with severe aortic stenosis: the group of patients in whom EF1 is most likely to be used. When limited to patients with a mean gradient of ≥40 mm Hg, a low EF1 was the only predictor of AVR or death after adjusting for mean gradient and NYHA class. Although this is a hypothesis-generating subgroup analysis, this finding is of relevance as there is substantial interest in optimising the timing of AVR in asymptomatic severe aortic stenosis, with several randomised controlled trials under way (NCT03042104, NCT02436655, NCT03094143).^21 22^ The study of EF1 in patients with low-gradient severe aortic stenosis—either low flow, paradoxical low flow or normal flow—would be valuable, but our numbers were insufficient to investigate this. Finally, all five deaths after AVR occurred in the group of 11 patients with a fixed low EF1, a finding that is in keeping with the poor long-term prognosis associated with the presence of LGE—representing irreversible replacement fibrosis—in aortic stenosis.^16–19^

### Strengths and limitations

Our study has several strengths. It is the first to establish the relationship between EF1 and both afterload and myocardial fibrosis, using a cohort enrolled in a multimodality imaging study and meticulously characterised with clinical assessment, echocardiography and CMR. The investigators measuring EF1 were blinded to all patient characteristics, CMR findings and outcomes, thus minimising bias. There are, however, several important limitations. First and foremost, this is a post hoc analysis of an observational cohort. EF1 was measured retrospectively and was unmeasurable in 10% of patients. While this proportion is similar to previously published data,^4^ all baseline echocardiograms in this cohort were performed for research purposes by a dedicated research sonographer or cardiologist. In clinical practice, routine image quality may be inferior. Furthermore, EF1 performed as a single measurement does not account for beat-to-beat variation in atrial fibrillation, a weakness common to most haemodynamic measurements. There was a low prevalence of atrial fibrillation at baseline in this cohort (n=5). Although future study and application of EF1 may be most pertinent in patients with asymptomatic severe aortic stenosis, this was less than half of the present cohort as we aimed to investigate associations with other measures of myocardial disease in addition to prognosis. Additionally, our analyses examining post-AVR change in EF1 and the relationship between a fixed low EF1 and long-term mortality, although of interest and clinically plausible, are limited by a small sample size and modest event rate. Similarly, we had insufficient patients with both iECV and EF1 available pre-AVR and post-AVR (n=18) to conduct a meaningful analysis of how changes in these parameters are related, although previous studies have demonstrated a reduction in iECV following AVR.^11 23^

## Conclusion

EF1 quantifies early, impaired LV function in patients with aortic stenosis and is associated with global afterload and myocardial fibrosis. Changes in EF1 occur before reduction in overall ejection fraction is seen. EF1 has a robust threshold that is strongly associated with future AVR, independent of mean gradient. Further prospective study is required to investigate the incremental utility of this novel measure in aortic stenosis.

**Key questions**

**What is already known on this subject?**

- Preliminary data have reported that first-phase ejection fraction (EF1) is a predictor of aortic valve intervention, hospitalisation for heart failure and death from any cause in patients with asymptomatic aortic stenosis and preserved ejection fraction.

**What might this study add?**

- We show that EF1 is reduced in a large proportion of patients with preserved overall ejection fraction and is associated with global afterload and myocardial fibrosis. We also provide validation of the prognostic power of EF1 for aortic valve replacement/death, which is independent of mean gradient. We demonstrate that this prognostic power is observed in the subgroup of patients with a mean gradient >40 mm Hg, independent of New York Heart Association class. Finally, we describe the dynamic nature of EF1, with recovery seen after aortic valve replacement, although this is attenuated by the presence of infarction.

**How might this impact on clinical practice?**

- The current paradigm of aortic valve intervention in aortic stenosis, based largely on standard echocardiographic measures and qualitative symptom assessment, has recently become a topic of much study and debate. Better identification of patients who may benefit from earlier intervention is required, particularly those with myocardial disease. Our observations are hypothesis-generating only but provide a strong rationale for future investigation into the role of EF1 for this purpose.
