# Supplementary material for: Determinants and prognostic value of echocardiographic first-phase ejection fraction in aortic stenosis
Source: Heart. 2020 Apr 28;106(16):1236–43. doi: 10.1136/heartjnl-2020-316684 (PMC7418600; doi:10.1136/heartjnl-2020-316684)
Supplement: Supplementary data [file heartjnl-2020-316684supp001.pdf]

Supplementary Table 1 Baseline characteristics by EF1 tertile

	Overall	1st tertile (5.6-20.9%)	2nd tertile (21.0-28.6%)	3rd tertile (28.7-43.6%)	p value
n	149	50	49	50	
Age	70.0 [65.0, 76.0]	69.0 [66.0, 74.8]	72.0 [66.0, 78.0]	70.0 [57.8, 75.0]	0.42
Male sex	104 (69.8)	40 (80.0)	32 (65.3)	32 (64.0)	0.16
Hypertension	102 (68.5)	32 (64.0)	39 (79.6)	31 (62.0)	0.12
Hyperlipidaemia	67 (45.0)	26 (52.0)	18 (36.7)	23 (46.0)	0.31
Diabetes	21 (14.1)	5 (10.0)	6 (12.2)	10 (20.0)	0.32
Coronary artery disease	56 (37.6)	26 (52.0)	16 (32.7)	14 (28.0)	**0.032**
Systolic blood pressure (mmHg)	148.5 [137.0, 165.5]	145.0 [135.4, 161.4]	149.0 [137.5, 165.5]	153.0 [140.5, 170.4]	0.62
Diastolic blood pressure (mmHg)	84.0 [77.0, 92.0]	85.2 [78.0, 93.5]	85.0 [80.0, 92.0]	80.8 [76.0, 89.9]	0.20
NYHA					**0.01**
I	71 (47.7)	15 (30.0)	26 (53.1)	30 (60.0)	
II	49 (32.9)	19 (38.0)	18 (36.7)	12 (24.0)	
III	26 (17.4)	13 (26.0)	5 (10.2)	8 (16.0)	
IV	3 (2.0)	3 (6.0)	0 (0.0)	0 (0.0)	
AV Vmax (m/s)	3.8 [3.2, 4.3]	4.2 [3.9, 4.6]	3.5 [3.1, 4.0]	3.3 [2.6, 3.9]	**<0.001**
AV mean gradient (mmHg)	32.9 [20.7, 41.7]	41.0 [36.9, 47.1]	26.3 [20.0, 36.0]	23.0 [14.6, 35.2]	**<0.001**
AV area (cm2)	0.9 [0.7, 1.1]	0.8 [0.7, 0.9]	0.9 [0.7, 1.1]	1.1 [0.8, 1.3]	**<0.001**
Valvuloarterial compliance (mmHg/ml/m2)	4.0 [3.3, 4.4]	4.1 [3.4, 4.9]	4.1 [3.2, 4.5]	3.7 [3.3, 4.1]	0.07
Indexed LV mass (g/m2)	87.0 [73.0, 99.0]	95.0 [83.0, 102.0]	86.0 [70.0, 98.0]	80.5 [69.2, 90.0]	**0.004**
Indexed stroke volume (ml/m2)	47.0 [41.0, 54.0]	45.5 [38.8, 55.0]	47.0 [40.0, 53.0]	49.0 [42.2, 53.0]	0.48
Ejection fraction (%)	66.7 [63.0, 70.7]	67.1 [61.4, 71.6]	66.7 [64.1, 68.9]	66.2 [63.2, 71.4]	0.73
EF1 (%)	25.6 [17.7, 29.9]	14.6 [10.9, 17.6]	25.6 [24.4, 27.1]	31.2 [29.9, 34.4]	**<0.001**
Global longitudinal strain (%)	-17.9 [-20.1, -15.4]	-16.9 [-18.9, -14.4]	-18.1 [-20.4, -16.1]	-18.2 [-21.1, -15.7]	0.**04**
Native T1	1179.0 [1157.0, 1207.0]	1191.0 [1170.0, 1214.0]	1171.0 [1152.2, 1201.5]	1181.5 [1149.0, 1196.8]	0.09
ECV fraction (%)	27.6 [25.6, 29.1]	27.7 [25.5, 30.2]	27.7 [26.2, 29.0]	27.4 [25.5, 28.6]	0.60
iECV (ml/m2)	22.3 [18.7, 26.2]	24.3 [20.2, 27.7]	21.9 [16.9, 27.3]	20.4 [17.5, 23.4]	**0.008**
Infarct LGE	21 (14.1)	14 (28.0)	6 (12.2)	1 (2.0)	**0.001**
Non-infarct LGE	36 (24.2)	17 (34.0)	14 (28.6)	5 (10.0)	**0.01**
hs-cTnI (ng/L)	6.6 [3.6, 12.4]	10.0 [5.0, 25.2]	4.9 [3.7, 10.2]	4.4 [3.1, 8.1]	**0.002**
BNP (ng/L)	26.8 [12.4, 54.2]	29.4 [13.6, 76.2]	29.4 [15.6, 53.4]	22.6 [8.0, 47.9]	0.17

Abbreviations: EF1, first-phase ejection fraction; NYHA, New York Heart Association; AV, aortic valve; LV, left ventricular; ECV, extracellular volume; iECV, indexed extracellular volume; LGE, late gadolinium enhancement; hs-cTnI, high-sensitivity cardiac troponin I; BNP, brain natriuretic peptide.

Bing R, *et al. Heart* 2020; 106:1236–1243. doi: 10.1136/heartjnl-2020-316684

Supplementary Table 2 Univariable linear regression models for EF1

	Coefficient	95% CI	r^2	p value
Age per 10 years	-0.03	-0.12, 0.06	0.003	0.53
Male sex	-0.13	-0.35, 0.08	0.009	0.24
Hypertension	0.05	-0.17, 0.27	0.001	0.67
Ejection fraction (log_2)	0.62	0.02, 1.22	0.027	**0.046**
Aortic valve gradient			0.256	**<0.001**
Mean gradient 20-39 mmHg	-0.18	-0.43, 0.07		
Mean gradient ≥40mmHg	-0.73	-0.95, -0.5		
Mean gradient (log_2)	-0.43	-0.55, -0.30	0.23	**<0.001**
Left ventricular mass index (log_2)	-0.51	-0.81, -0.21	0.07	**0.001**
Valvuloarterial compliance (log_2)	-0.34	-0.62, -0.06	0.038	**0.017**
Native T1 (log_2)	-2.35	-4.39, -0.32	0.035	**0.025**
Extracellular volume fraction (log_2)	-0.61	-1.38, 0.16	0.017	0.12
Indexed extracellular volume (log_2)	-0.45	-0.70, -0.19	0.074	**0.001**
Late gadolinium enhancement (any)	-0.41	-0.61, -0.20	0.092	**<0.001**
Global longitudinal strain (log_2)	-0.27	-0.48, -0.06		**0.013**
High-sensitivity cardiac troponin I (log_2)	-0.08	-0.14, -0.01	0.038	**0.019**
Brain natriuretic peptide (log_2)	-0.06	-0.13, 0.01	0.021	0.10

Bing R, *et al. Heart* 2020; 106:1236–1243. doi: 10.1136/heartjnl-2020-316684

Supplementary Table 3 Baseline characteristics in those who had AVR stratified by fixed low baseline EF1 (<25%)

	Fixed	Non-fixed	p value
n	11	46	
EF1 baseline (%)	18.5 [13.2, 22.2]	15.6 [12.0, 26.4]	0.98
EF1 post-AVR (%)	14.6 [12.6, 23.2]	28.1 [23.2, 32.3]	**<0.001**
Baseline echocardiogram to AVR (days)	159.0 [67.0, 649.0]	198.5 [63.8, 1051.5]	0.97
AVR to follow-up echocardiogram (days)	386.0 [264.5, 464.0]	340.5 [165.2, 389.8]	0.21
Age	74.0 [66.5, 76.0]	70.5 [65.0, 74.8]	0.39
Male sex	9 (81.8)	35 (76.1)	0.99
Hypertension	9 (81.8)	31 (67.4)	0.57
Hyperlipidaemia	6 (54.5)	23 (50.0)	1.00
Diabetes	1 (9.1)	4 (8.7)	1.00
Coronary artery disease	7 (63.6)	21 (45.7)	0.46
Systolic blood pressure (mmHg)	144.0 [138.5, 154.2]	143.0 [133.5, 163.8]	0.69
Diastolic blood pressure (mmHg)	84.5 [78.5, 92.5]	85.0 [77.0, 92.0]	0.94
NYHA			0.08
I	3 (27.3)	17 (37.0)	
II	6 (54.5)	16 (34.8)	
III	1 (9.1)	13 (28.3)	
IV	1 (9.1)	0 (0.0)	
AV Vmax (m/s)	4.0 [3.7, 4.5]	4.2 [3.9, 4.6]	0.20
AV mean gradient (mmHg)	37.7 [28.0, 44.8]	40.7 [36.4, 45.3]	0.28
AV area (cm2)	0.9 [0.7, 0.9]	0.8 [0.7, 0.9]	0.45
Valvuloarterial compliance (mmHg/ml/m2)	3.8 [3.5, 4.0]	4.0 [3.4, 4.9]	0.59
Indexed LV mass (g/m2)	95.0 [84.0, 100.8]	92.5 [80.2, 102.0]	0.84
Indexed stroke volume (ml/m2)	49.0 [47.2, 53.9]	48.0 [41.0, 55.0]	0.65
Ejection fraction (%)	65.6 [53.6, 68.9]	67.1 [65.6, 72.0]	0.20
Global longitudinal strain (%)	-18.2 [-19.0, -13.6]	-17.3 [-18.7, -15.5]	0.90
Native T1	1179.0 [1163.0, 1206.5]	1191.0 [1165.0, 1207.0]	0.61
ECV fraction (%)	27.8 [26.7, 28.8]	26.9 [25.3, 28.5]	0.49
iECV (ml/m2)	22.8 [21.2, 27.3]	23.4 [19.5, 25.5]	0.63
Infarct LGE	7 (63.6)	4 (8.7)	**<0.001**
Non-infarct LGE	1 (9.1)	11 (23.9)	0.50
hs-cTnI (ng/L)	10.0 [7.1, 13.8]	6.7 [4.3, 13.7]	0.44
BNP (ng/L)	28.1 [20.5, 116.0]	25.6 [13.1, 64.8]	0.16

Abbreviations: EF1, first-phase ejection fraction; NYHA, New York Heart Association; AV, aortic valve; LV, left ventricular; ECV, extracellular volume; iECV, indexed extracellular volume; LGE, late gadolinium enhancement; hs-cTnI, high-sensitivity cardiac troponin I; BNP, brain natriuretic peptide.

Bing R, *et al. Heart* 2020; 106:1236–1243. doi: 10.1136/heartjnl-2020-316684

Supplementary Table 4 Univariable logistic regression models for fixed low EF1 after AVR

	**Coefficient**	**95% CI**	**Pseudo-r^2**	**p value**
Age per 10 years	0.36	-0.42, 1.14	0.02	0.36
Male sex	0.35	-1.33, 2.02	0.00	0.69
Hypertension	0.78	-0.87, 2.43	0.03	0.36
Coronary artery disease	0.73	-0.62, 2.09	0.03	0.29
Ejection fraction (log_2)	-3.03	-6.17, 0.11	0.10	0.06
Mean gradient (log_2)	-1.10	-2.91, 0.71	0.04	0.23
Left ventricular mass index (log_2)	-0.07	-2.26, 2.11	0.00	0.95
Valvuloarterial compliance (log_2)	-0.35	-2.13, 1.42	0.00	0.70
Native T1 (log_2)	-8.56	-20.58, 3.47	0.06	0.16
Extracellular volume fraction (log_2)	0.85	-4.08, 5.78	0.00	0.74
Indexed extracellular volume (log_2)	0.08	-1.69, 1.85	0.00	0.93
Late gadolinium enhancement (infarct)	2.91	1.31, 4.51	0.36	**<0.001**
Late gadolinium enhancement (non-infarct)	-1.15	-3.31, 1.02	0.04	0.30
Global longitudinal strain (log_2)	0.55	-1.08, 2.18	0.01	0.51
High-sensitivity cardiac troponin I (log_2)	0.03	-0.38, 0.43	0.00	0.90
Brain natriuretic peptide (log_2)	0.45	-0.08, 0.98	0.1	0.10

Bing R, *et al. Heart* 2020; 106:1236–1243. doi: 10.1136/heartjnl-2020-316684
