# Supplementary figures and images for: Determinants and prognostic value of echocardiographic first-phase ejection fraction in aortic stenosis
Source: Heart. 2020 Apr 28;106(16):1236–43. doi: 10.1136/heartjnl-2020-316684 (PMC7418600; doi:10.1136/heartjnl-2020-316684)

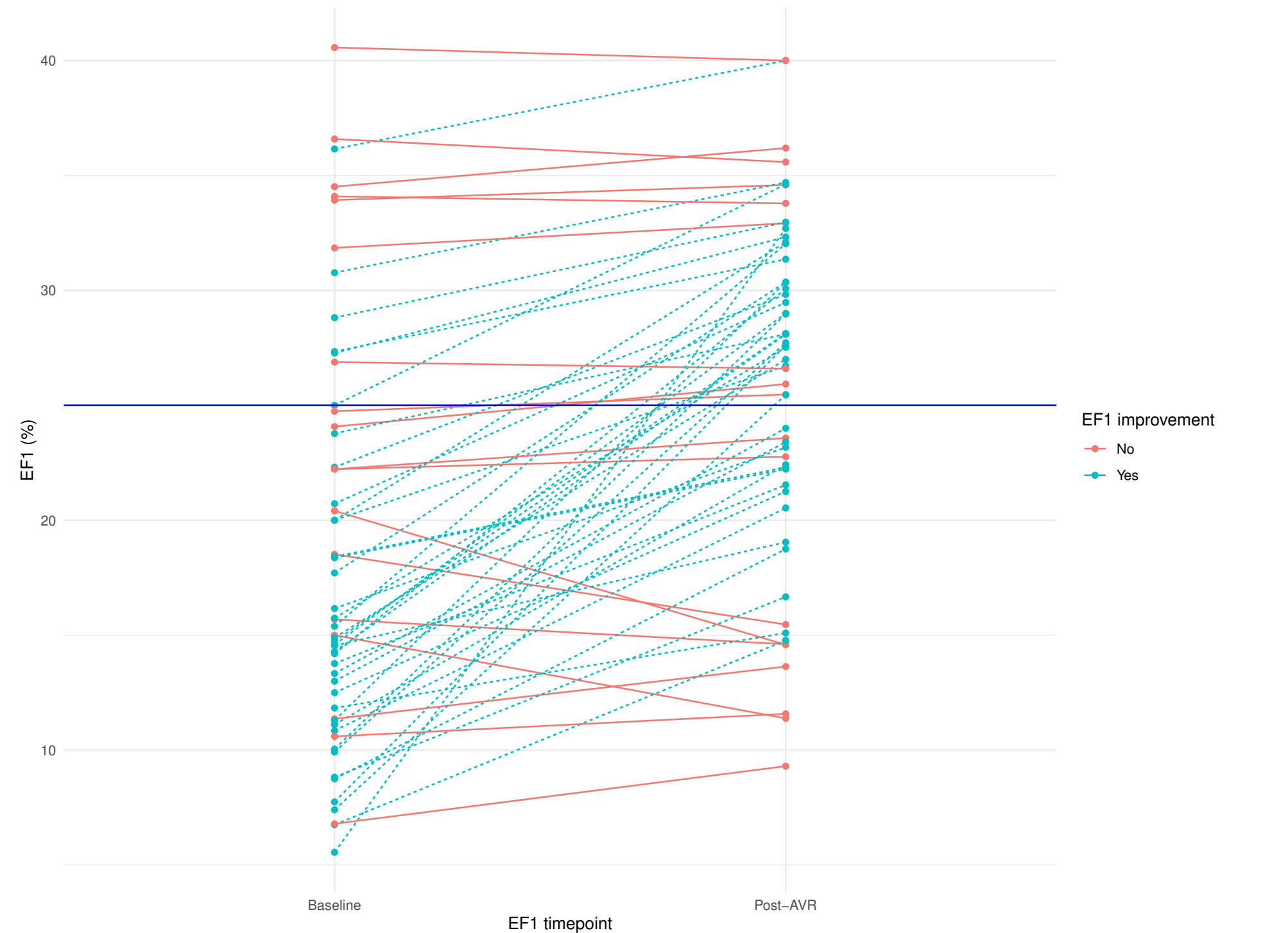

Supplement: Supplementary data [file heartjnl-2020-316684supp002.pdf]

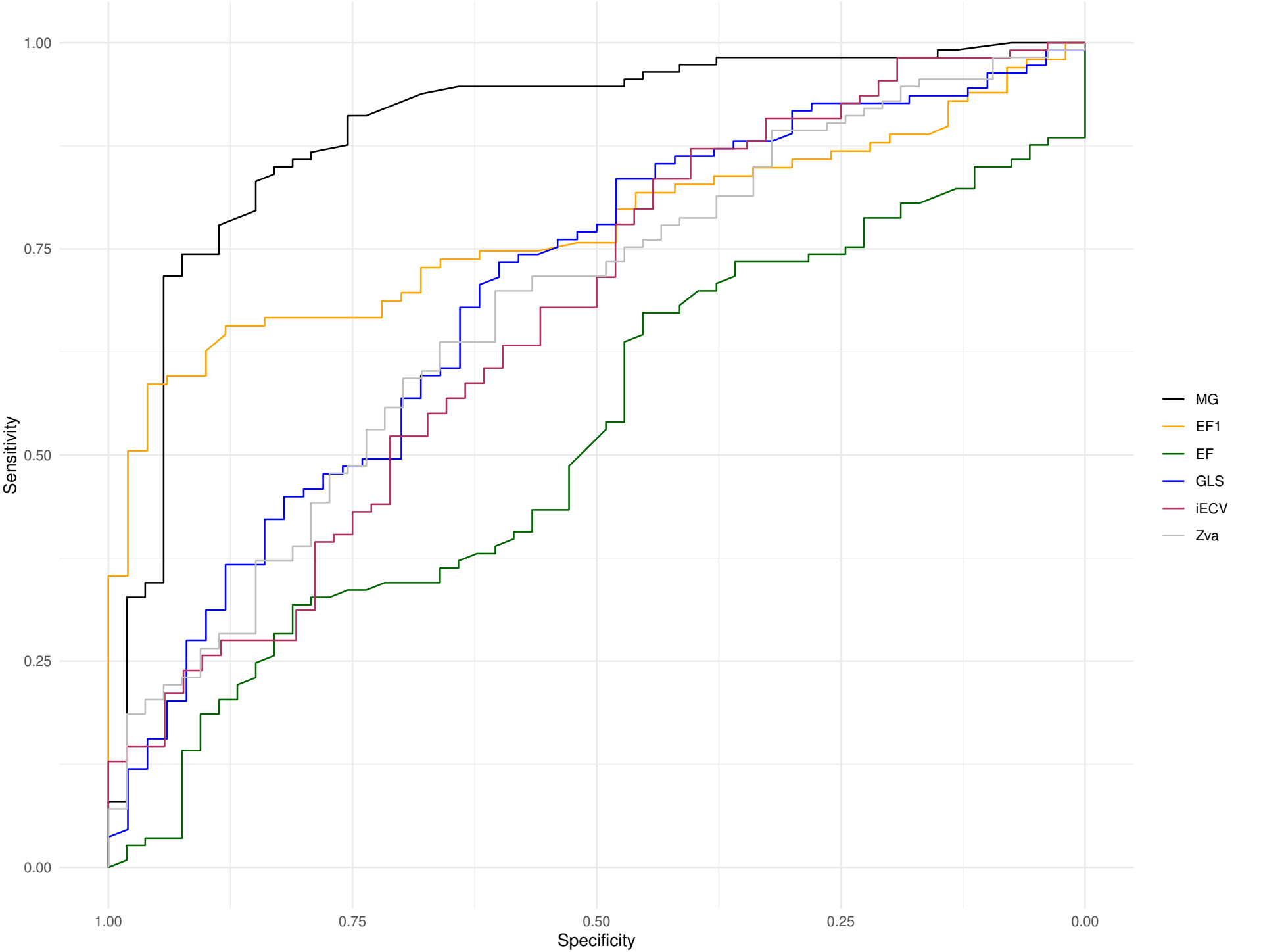

Supplement: Supplementary data [file heartjnl-2020-316684supp003.pdf]

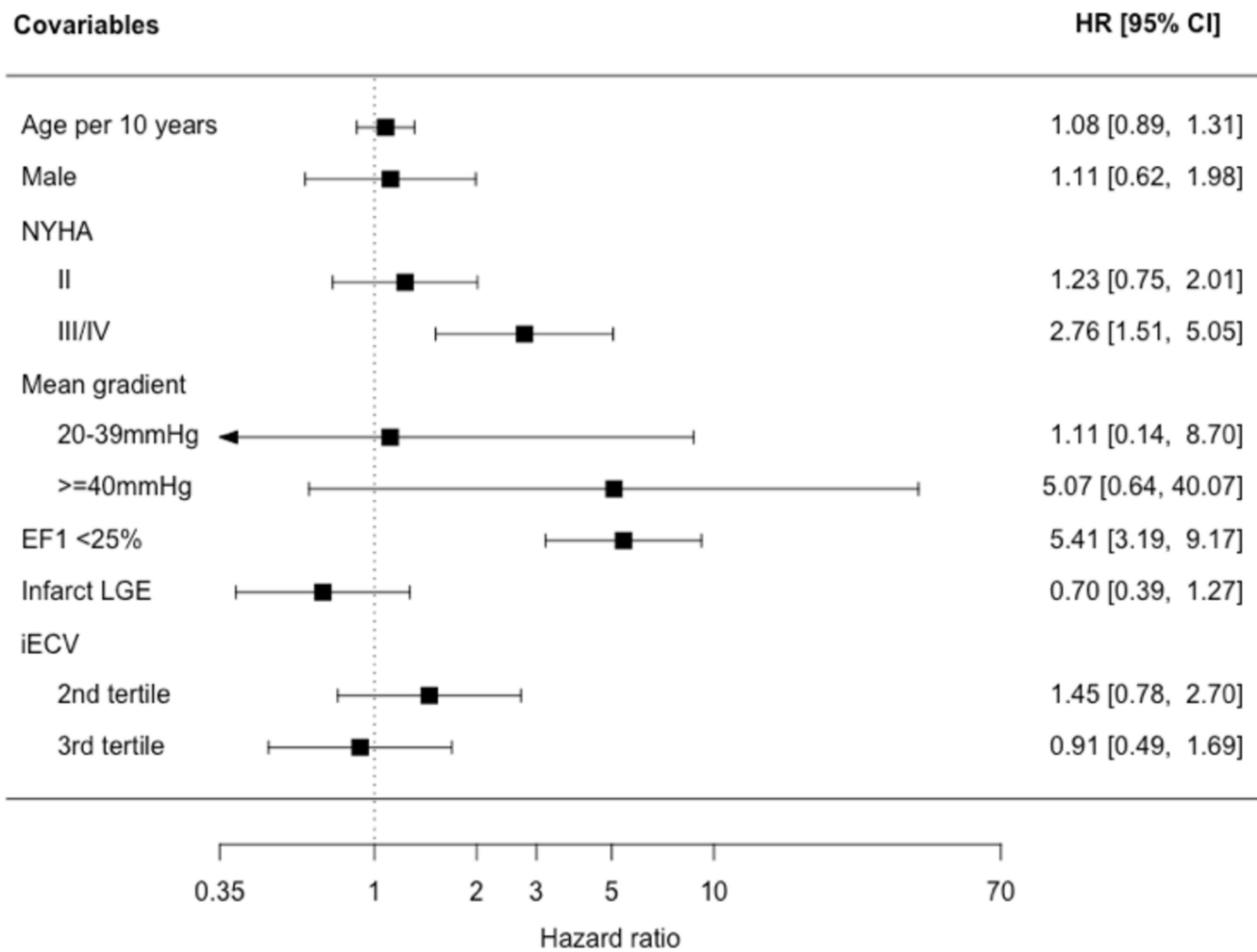

Supplement: Supplementary data [file heartjnl-2020-316684supp004.pdf]
